# Classification of Prostate Transitional Zone Cancer and Hyperplasia Using Deep Transfer Learning From Disease-Related Images

**DOI:** 10.7759/cureus.14108

**Published:** 2021-03-25

**Authors:** Bo Hu, Lin-Feng Yan, Yang Yang, Ying Yu, Qian Sun, Jin Zhang, Hai-Yan Nan, Yu Han, Yu-Chuan Hu, Ying-Zhi Sun, Gang Xiao, Qiang Tian, Cui Yue, Jia-Hao Feng, Liang-Hao Zhai, Di Zhao, Guang-Bin Cui, Valerie Lockhart Welch, Elyse M Cornett, Ivan Urits, Omar Viswanath, Giustino Varrassi, Alan D Kaye, Wen Wang

**Affiliations:** 1 Department of Radiology, Fourth Military Medical University, Shaanxi, CHN; 2 Department of Computer Network Information, Chinese Academy of Science, Beijing, CHN; 3 Department of Pathology, Louisiana State University (LSU) Health Shreveport, Shreveport, USA; 4 Department of Anaesthesiology, Louisiana State University (LSU) Health Shreveport, Shreveport, USA; 5 Department of Anesthesia, Critical Care and Pain Medicine, Beth Israel Deaconess Medical Center, Harvard Medical School, Boston, USA; 6 Department of Pain Management, University of Arizona, Phoenix, USA; 7 Department of Research, Paolo Procacci Foundation, Roma, ITA; 8 Department of Anesthesiology, Louisiana State University Health Shreveport, Shreveport, USA

**Keywords:** deep learning, transfer learning, magnetic resonance imaging (mri), prostate cancer

## Abstract

Purpose

The diagnosis of prostate transition zone cancer (PTZC) remains a clinical challenge due to their similarity to benign prostatic hyperplasia (BPH) on MRI. The Deep Convolutional Neural Networks (DCNNs) showed high efficacy in diagnosing PTZC on medical imaging but was limited by the small data size. A transfer learning (TL) method was combined with deep learning to overcome this challenge.

Materials and methods

A retrospective investigation was conducted on 217 patients enrolled from our hospital database (208 patients) and The Cancer Imaging Archive (nine patients). Using T2-weighted images (T2WIs) and apparent diffusion coefficient (ADC) maps, DCNN models were trained and compared between different TL databases (ImageNet vs. disease-related images) and protocols (from scratch, fine-tuning, or transductive transferring).

Results

PTZC and BPH can be classified through traditional DCNN. The efficacy of TL from natural images was limited but improved by transferring knowledge from the disease-related images. Furthermore, transductive TL from disease-related images had comparable efficacy to the fine-tuning method. Limitations include retrospective design and a relatively small sample size.

Conclusion

Deep TL from disease-related images is a powerful tool for an automated PTZC diagnostic system. In developing regions where only conventional MR scans are available, the accurate diagnosis of PTZC can be achieved via transductive deep TL from disease-related images.

## Introduction

About 25% of prostate cancers originate in the transition zone (TZ), and their diagnosis remains a clinical challenge due to the similarity on MRI to benign prostatic hyperplasia (BPH) [1]. The conventional transrectal ultrasound-guided biopsy faces the dilemma of both underdiagnosis and overdiagnosis because it is invasive, the tumor may in fact be small, and there are inherent limitations of H&E slides [2]. Additionally, in developing areas, screening efficacy is impaired by the lack of radiology interpretation expertise. Consequently, several machine learning methods have been developed to classify prostate cancer from normal tissue or BPH [3].

Traditional machine learning methods are laborious because of complex feature extraction procedures [4]. Most importantly, the selection of features may be influenced by different data sources and processing software, thus its generalization is limited. The Deep Convolutional Neural Networks (DCNNs) automatically extract medical imaging diagnostic features based on fixed architectures [5,6]. Furthermore, several deep learning studies have been based on multi-center databases, which proved the robustness of the DCNN model [7,8]. The DCNN model is a data-dependent classifier, where a larger database yields a better result. However, prostate TZ cancer (PTZC) images are scarce [1]. DCNNs were developed to imitate how the visual cortex of the brain processes and recognizes images, but the learning procedure has always been regarded as a “black box” [9]. Clinicians can learn one action much easier and faster if they had learned similar things before, called transfer learning (TL). In machine learning, TL is designed to transfer the information from a particular source domain to the target domain [10,11]. TL can combine with deep learning to overcome the issue of small sample size [12,13]. By transferring similar features from everyday pictures to disease states, previous DCNN studies showed TL's efficacy based on a large natural image database, ImageNet [7,14,15].

However, a more effective way for clinicians to learn one disease is to learn a different yet similar disease analogically. The imaging manifestation of PTZC is similar to prostate peripheral zone cancer (PPZC) in some ways, and there are far more images of PPZC available [16]. Consequently, to deal with the scarcity of PTZC images and better differentiate them from BPH, we decided to imitate the human brain's analogical learning ability by combining TL and DCNN. In this current study, we trained DCNN models and compared varied TL databases [ImageNet (natural images) vs. PPZC images (disease-related images)] and protocols (scratch, fine-tuning, or transductive transferring).

This article was previously posted to the Research Square preprint server on July 20, 2020.

## Materials and methods

Patients

**Figure 1:**
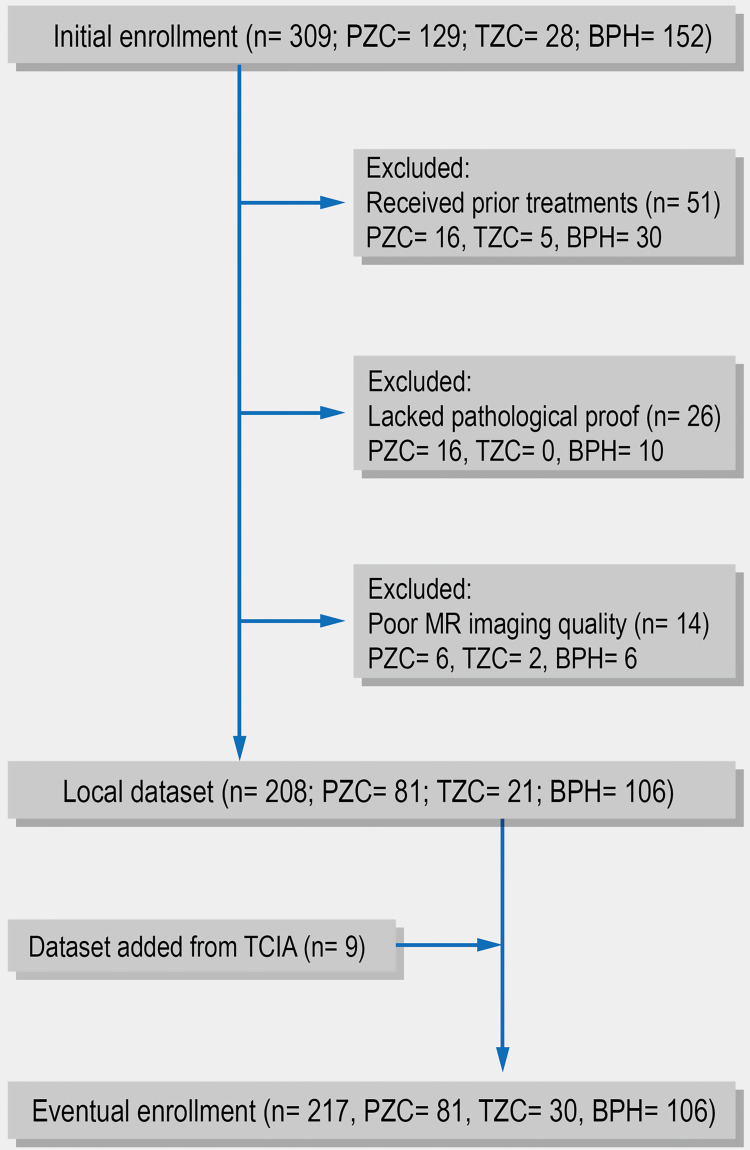
Patients' recruiting procedure PZC = peripheral zone cancer, TZC = transitional zone cancer, BPH = benign prostatic hyperplasia.

From May 2010 to March 2016, the detailed clinical information and MRI images of 309 patients from our hospital were retrospectively recruited. After excluding patients who received previous surgery or medication, lacked pathologic diagnosis, or had poor MRI qualities, 208 pathologically confirmed prostate cancer or BPH patients were enrolled in the current study. These patients underwent a series of MR scanning, followed by radical prostatectomy or MRI-guided biopsy within a month. A Gleason score greater than or equal to six was considered a malignant tumor. Clinical data from the 208 patients from the local dataset was collected and is shown in Table 1. Nine PTZC patients from The Cancer Imaging Archive (TCIA) [17] were also enrolled, resulting in a final enrollment of 217 patients (Figure 1).

**Table 1:** Basic information (standard deviation) of patients of the local dataset. PCA = prostate cancer; BPH = benign postate hypertrophy; F-PSA = free prostate specific antigen; T-PSA = total prostate specific antigen

Clinical features	PCA	BPH	P-value
Age	70.44 (±9.73)	70.07 (±7.94)	0.73
Weight	67.72 (±10.60)	67.58 (±10.86)	0.92
Blood Glucose	5.68 (±0.95)	5.65 (±1.78)	0.84
F-PSA	24.00 (42.93)	3.50 (5.50)	<0.001
T-PSA	387.58 (837.62)	21.06 (45.00)	<0.001
F/T	0.12 (0.44)	0.18 (0.08)	<0.001

Imaging data

MRI protocols were implemented on three 3T (GE DISCOVERY MR750, GE SIGNA EXCITE, and MAGNETOM Skyra) and two 1.5T scanners (GE SIGNA HDxt and SIEMENS Area). All parameters of the local scanner were provided in Table 2 (Appendix). Patients had received axial T2-weighted images (T2WIs) and axial multiple b-value DWI (multi-b DWI) scans. Apparent diffusion coefficient (ADC) maps were calculated by using two different b values (0 and 1,000 s/mm^2^). Images from TCIA included axial T2WIs and axial ADC maps.

Preprocessing

**Figure 2:**
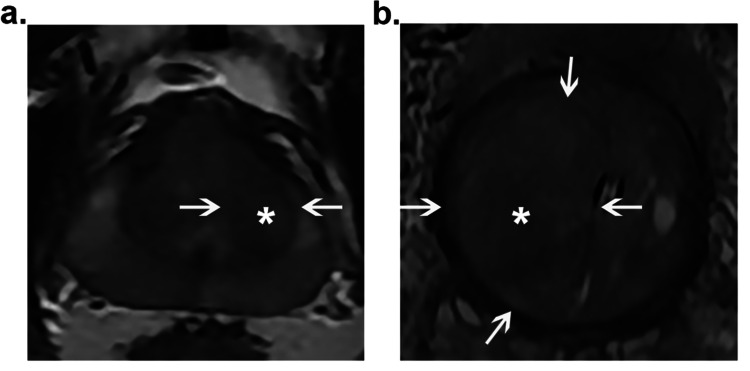
The confirmation process of tumor location. (a) A 60-year-old man with mild urinary symptoms. This patient underwent MR imaging and was diagnosed with cancer after prostatectomy. (b) A 67-year-old man with severe urinary symptoms. This patient underwent an MRI-guided biopsy and was diagnosed with benign prostatic hyperplasia.

Images were converted from DICOM to bitmap format. The locations of individual lesions on the MR image were independently determined by two radiologists with 15 years of experience (C. Y. and YZ. S.) (Figure 2). After these two experienced radiologists read all three modalities of the images from one patient, range of interests (ROIs) were drawn by hand on all modalities and planes where the tumor is visible. After that, the target lesion was cropped with a rectangular ROI, located in the center of the image and occupying 80% of the area. These cropped images were then resized to a 256 × 256 matrix using a bilinear interpolation method.

**Figure 3:**
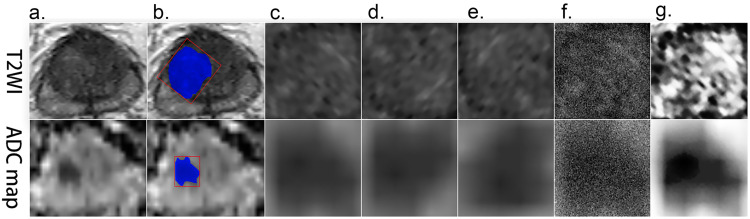
Preprocessing procedure of images of three protocols. (a) Source images. (b) The area needing to be cropped. (c) The resized cropped area. (d) Rotation by 90 degrees. (e) Vertical flipping. (f) Adding Gaussian noise. (g) Contrast enhancement by histogram equalization.

Data augmentation plays a vital role in the utilization of DCNN, and it can significantly improve the efficacy of a DCNN classifier [18]. Images were augmented using random cropping, mean subtraction, and mirror images, which were prebuilt options within the Caffe framework. Further augmentation included 90°-rotation, vertical flipping, adding standard Gaussian white noise, and histogram equalization processed using MATLAB (Matrix Laboratory 2016b, Mathworks, Natick, MA) [19]. Since some of these processed images are intrinsically different from the original images, test sets were not augmented all through the project to prevent biases. The preprocessing procedure is described in Figure 3.

Training procedure

**Figure 4:**
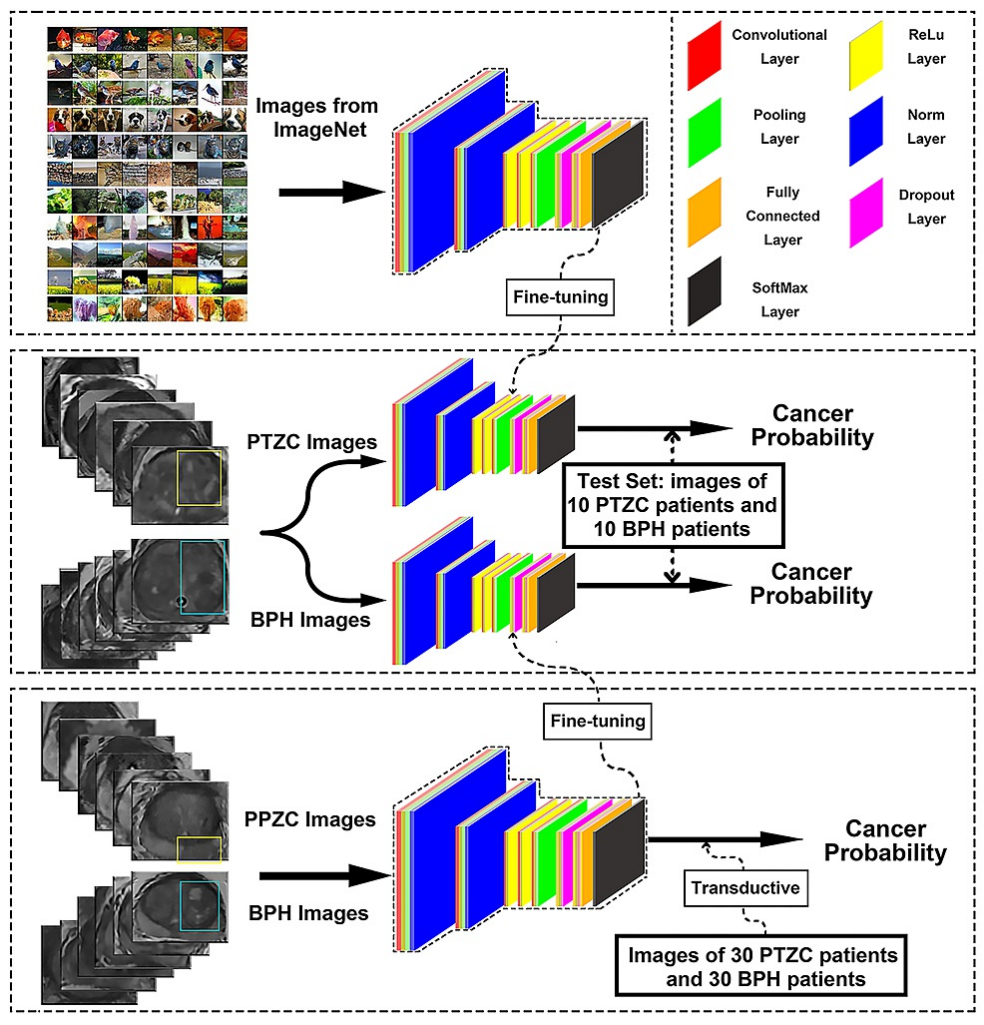
Procedure of TL. Feature extraction would be conducted with “weights” of the network. After dozens or hundreds of times of training, these “weights” would be optimized. TL was conducted by transferring the adjustable weights from the model trained with either data from disease-related domain or ImageNet to our target domain. ReLu = rectified-linear activation

Prostate images were processed via a series of operations to produce a predictive probability for each image Figure 4. DCNNs include various layers to form a pipeline of extraction, pooling, nonlinear processing, imaging feature synthesis, and final probability output of these class labels (malignancy vs. hyperplasia in the current study). These procedures were conducted with the “weights” in the whole network, which were randomly initialized before the training procedure. A DCNN is trained to discover and optimize the “weights” via a back-propagation process. After dozens or hundreds of training, an optimized set of “weights” could be obtained to exert a sufficient predicting ability [18].

Of the 106 BPH patients, 30 BPH patients were randomly selected as the counterpart of the 30 PTZC patients. About 400 images of the 60 selected patients were defined as our target dataset to train and test an Alex-Net DCNN

TL procedure

TL can improve a classifier in one domain by transferring knowledge from a larger relevant domain. In some cases, this goal can be achieved by using the “off-the-shelf” “weights” trained with data from the relevant domain. The transferred “weights” could be used directly to classify the target data, a process often called transductive TL [16]. In other cases, the “weights” of the network are retrained with the target data, but under a lower base learning rate, a process often called “fine-tuning” [17]. These two methods were tested in the current study, and both an Alex-Net and a Google-Net were trained.

Two models were pre-trained for TL. One was about 900 images of 81 PPZC and 76 BPH, and the other was the ImageNet containing 1.2 million natural color images [20].

Statistical analysis

For each image, the final output was defined as the probability that the lesion is malignant. On the test datasets, receiver operating characteristic (ROC) curves were plotted according to these output values, and the area under the curves (AUCs) and their 95% confidence intervals (CI) were determined [21]. For these ROC curves, comparisons between AUCs were made using the DeLong and Clarke-Pearson method [22], and P values less than 0.05 were considered statistically significant. When it came to multiple comparison tests, P values were corrected using a post hoc Bonferroni method [23]. The optimal accuracy, sensitivity, and specificity were determined from the optimal cutoff value by the Youden Index (YI) according to the following equation: YI = [1 - (sensitivity + sensitivity)]. Statistical analysis and graph creation were completed in Sigmaplot 12.5 software (Systat Software, Inc, Point Richmond, CA).

## Results

PTZC and BPH could be classified with Alex-Net DCNN

**Figure 5:**
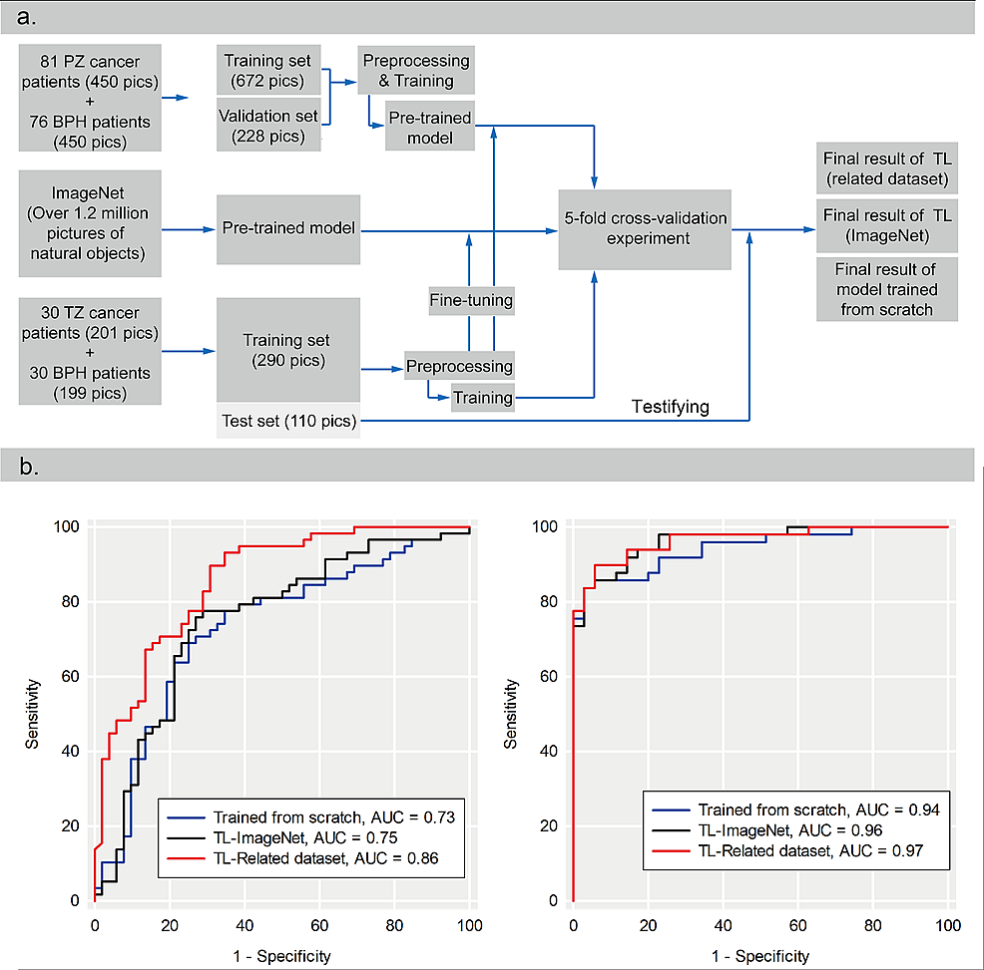
Experiment on the efficacy of traditional deep learning method and the TL method. (a) The flow chart of data grouping and model training and evaluating. (b) Comparison of ROC curves for the model trained from scratch and trained by TL. TL - transfer learning TL-ImageNet = trained by transferring information from ImageNet. TL-Related dataset = trained by transferring information from the related dataset.

The 60 patients in the target dataset were randomly divided into a test set (20 patients, 110 pictures) and a training set (40 patients, 290 pictures). Then, five Alex-net models were trained through a five-fold cross-validation method. In each training procedure, 4/5 of the data in the training set was used to train the model, while the remaining 1/5 was used as the validation set to select the optimal model. After that, the test set was used to test the efficacy of those five models, and for each image, five probabilities were derived. In the end, five probabilities of each image were averaged to calculate the final output value (Figure 5a).

Even with the small sample size, prostate cancer can be distinguished using a DCNN model (Figure 5b). Using the model trained from scratch (TFS-model), T2WIs were associated with an AUC of 0.73 (95% CI = 0.63-0.83) and a sensitivity, specificity, and accuracy of 69%, 75%, and 81%, respectively. ADC images were associated with an AUC of 0.94 (95% CI = 0.90-0.99) and a sensitivity, specificity, and accuracy of 84%, 97%, and 89%, respectively. The diagnostic efficacy of Alex-Net DCNN model using ADC images was quite satisfying, but that using T2WI needed to be improved further.

The performance of TL from natural pictures (ImageNet) is limited by the small data size

Using a similar protocol and Alex-Net, the DCNN model was pre-trained with 1.2 million natural color pictures from ImageNet and the performance of TL was investigated (Figure 5b).

Using the model trained from ImageNet (TFI-model), T2WIs were associated with an AUC of 0.75 (95% CI = 0.65-0.84) and sensitivity, specificity, and accuracy of 76%, 73%, and 75%, respectively. ADC images were associated with an AUC of 0.96 (95% CI = 0.90-0.99) and sensitivity, specificity, and accuracy of 84%, 97%, and 89%, respectively. TL from natural images resulted in slight improvement of efficacy based on T2WIs (P = 0.17) and ADC images (P = 0.07).

TL from disease-related images (PTZC images) improved the diagnostic efficacy of DCNN model

The DCNN model was pre-trained with the remaining 76 BPH and 81 PPZC images, randomly divided into a training set (122 patients, 672 pictures, for model training) and a validation set (35 patients, 228 pictures, for optimal model selecting). This pre-trained model was fine-tuned with the aforementioned target dataset (PTZC and BPH images, Figure 5b).

Using the model trained from the disease-related dataset (TLR-model), T2WIs were associated with an AUC of 0.86 (95% CI = 0.79-0.93) and sensitivity, specificity, and accuracy of 90%, 69%, and 80%, respectively. The diagnostic efficacy of the TLR-model was significantly higher than that of the TFS-model (P = 0.00014) and the TLI-model (P = 0.00046). ADC images were associated with an AUC of 0.97 (95% CI = 0.90-0.99) and sensitivity, specificity, and accuracy of 90%, 94%, and 92%, respectively. However, there was no significant difference between AUCs of the TLR-model and the TLI-model (P = 0.88) or the TLR-model and the TFS-model (P = 0.29).

The transductive method is a novel and effective way for TL

**Figure 6:**
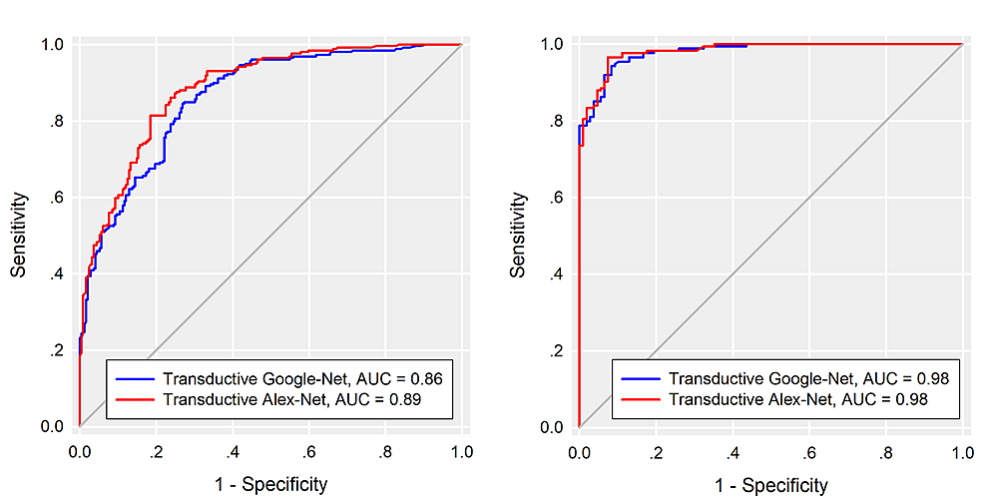
(a) AUCs of transductive TL Alex-Net and Google-Net models, T2WI protocol. (b) AUCs of transductive TL Alex-Net and Google-Net models, ADC maps. AUC - area under the curves T2WI - T2-weighted image ADC - apparent diffusion coefficient

A transductive Google-Net and a transductive Alex-Net model were trained with images of PPZC and BPH, and the models were directly employed on all PTZC and BPH images. ROC curves and the AUCs were obtained (Figure 6).

Using the transductive Google-Net model, T2WIs were associated with an AUC of 0.86 (95% CI = 0.83-0.89) and sensitivity, specificity, and accuracy of 84%, 73%, and 79%, respectively. ADC images were associated with an AUC of 0.98 (95% CI = 0.97-0.99) and sensitivity, specificity, and accuracy of 94%, 92%, and 93%, respectively. Using the transductive Alex-Net model, T2WIs were associated with an AUC of 0.89 (95% CI = 0.86-0.91) and sensitivity, specificity, and accuracy of 81%, 82%, and 81%, respectively. ADC images were associated with an AUC of 0.98 (95% CI = 0.97-0.99) and sensitivity, specificity, and accuracy of 97%, 93%, and 95%, respectively.

Ensemble contributes to the stabilization of output values

Because each lesion may have two modalities and multiple planes, ensembles were performed by averaging these planes' output values to get a stable predicting output for each target lesion. Scatter plots were made using these averaged values. Statistical analysis and graph creation were completed in Sigmaplot 12.5 software (Systat Software, Inc, Point Richmond, CA).

**Figure 7:**
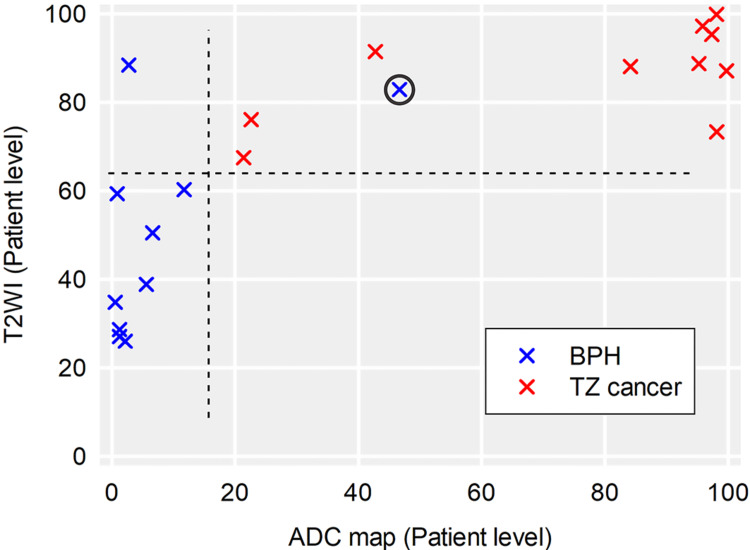
Results of ensemble. Ensemble was conducted by averaging all slices of same target lesions. On the patient level, each dot refers to the output value of a target lesion. The result showed that ensemble is a powerful method to eliminate variations and can help to generate a stable output value. Only one patient (circled) was misdiagnosed in both protocols.

**Figure 8:**
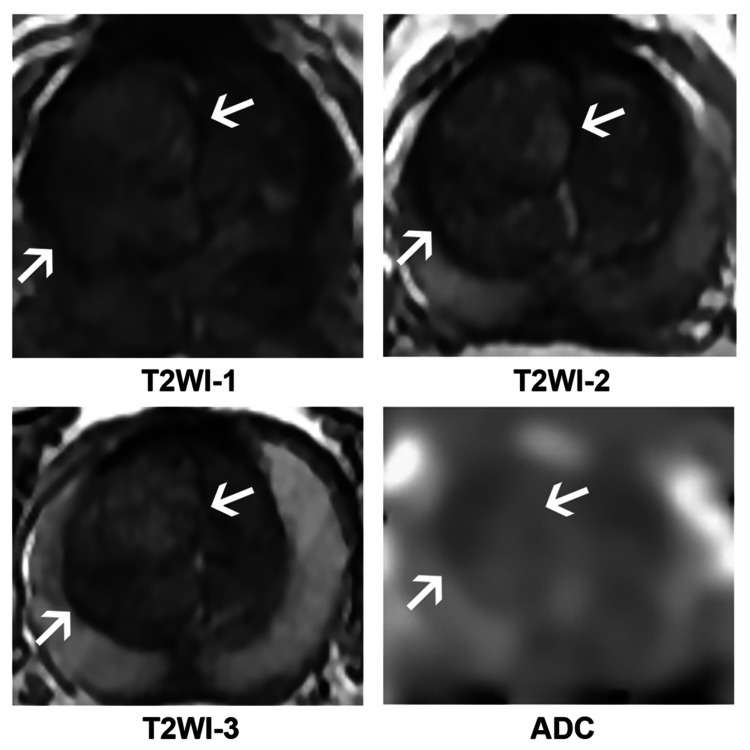
T2WIs and ADC map of the patient misdiagnosed in both protocols after the ensemble procedure

The results of the ensemble are shown in Figure 7, which revealed that the averaging method could largely eliminate variations among images of different slices and help generate a stable output. Of these 20 patients, two patients were misdiagnosed with T2WIs, while only one patient was misdiagnosed with ADC images (Figure 8).

## Discussion

Based on the current investigation, we revealed that PTZC and BPH can be distinguished through traditional DCNN. The efficacy of TL from natural pictures to T2WI and ADC images was limited, but TL from the disease-related images significantly improved the diagnostic efficacy of T2WI. Transductive TL from disease-related images had similar diagnostic efficacies to the fine-tuning method on both T2WI and ADC images. We also found that the DCNN model is robust enough to process images from different sources.

The efficacy of TL from natural images was significant in previous studies, but our findings contradicted theirs [7,13,24]. In our study, the diagnostic efficacy was improved by TL from natural images, but the improvement was not significant. This may be because the DCNN model had learned useful texture characteristics from natural images and applied them to the diagnosis of PTZC. However, the efficacy was still limited with the small sample size of rare disease and/or the incapability of the shallow Alex-Net model. On the contrary, the DCNN model trained with disease-related images performed significantly better. Therefore, the DCNN model seems similar to the human brain neural network in that learning directly from a related disease is more effective. There were also studies that focused on transductive TL, but to our knowledge, very few studies had applied this method on medical images [25-27]. It is imperative to test the applicability of transductive DCNN for diseases before it can evolve from the bench to the bedside. In the future, this TL method could be used to diagnose rare diseases, such as differentiating lung cancer lymphatic spread from normal lymphatic tissue by transferring information from lung cancer.

Previous research suggests that DCNN is effective in classifying prostate cancer and BPH or other benign lesions, but very few studies were specifically conducted to classify PTZC and BPH [28-30]. This issue is important because the diagnostic accuracy between PTZC and BPH in previous studies may be biased due to a high number of PPZC patients. These studies also revealed that T2WI and ADC map are the two most efficient protocols. We revealed that the diagnostic efficacy of T2WI was lower than ADC images with a small sample size, which was partly made up by TL. As a result, we assumed that although features of ADC images were simple and effective, the potential of T2WI could be tapped more fully by experienced radiologists. In developing regions where advanced MRI is lacking, the full utilization of T2WIs using the current TL-DCNN strategy could be a critical and practical way to improve diagnostic efficacy.

Our study has two limitations. First, although we applied a TL method to make up for the shortcomings caused by the small sample size, it still had limited help for practical applications. Second, because of the retrospective nature of the current study, some bias cannot be ruled out. Thus a randomized controlled trial (RCT) should be conducted in the future.

## Conclusions

The diagnostic efficacy of the Alex-Net model for differentiating PPZC and BPH could be significantly improved by transferring the disparity information between PTZC and BPH, which was better than TL from ImageNet. Furthermore, transductive TL model trained with the data of PPZC and BPH could be directly used to classify PTZC from BPH. This study has evidenced several important aspects that may help in a more advanced understanding of the full topic. For sure, further specific studies would increase the knowledge, which would be extremely helpful in a clinical setting of wide interest.

## Appendices

**Table 2:** Table S1. Detailed imaging parameters of four scanners. (A) GE Discovery MR 750. (B) GE EXCITE. (C) GE Hdxt. (D) SIEMENS Area. DWI - diffusion-weighted imaging; TR - repetition time; TE - echo time; FOV - field of view

MR 750	T2	T2FS	DWI (b = 0)	DWI (b = 1,000)
TR	4,751	5,711	3,600	3.600
TE	122	89	64	64
MATRIX	512 x 512	512 x 512	256 x 256	256 x 256
FOV	240	200	200	200
Slices Thickness	3.5	4	4	4
Acquisition Time	180	176	181	181
Flip Angle	90	90	90	90

GE EXCITE	T2	T2FS	DWI (b = 0)	DWI (b = 1,000)
TR	4,200	2,300	1,600	1,600
TE	131	98	52	52
MATRIX	512 x 512	512 x 512	256 x 256	256 x 256
FOV	240	200	200	200
Slices Thickness	4	4	4	4
Acquisition Time	90	85	91	91
Flip Angle	90	90	90	90

GE Hdxt	T2	T2FS	DWI (b = 0)	DWI (b = 1,000)
TR	3,415	3,367	3,600	3,600
TE	125	72	58	58
MATRIX	512 x 512	512 x 512	256 x 256	256 x 256
FOV	240	200	200	200
Slices Thickness	4	4.5	4.5	4.5
Acquisition Time	111	110	111	111
Flip Angle	90	90	90	90

SIEMENS Area	T2	T2FS	DWI (b = 0)	DWI (b = 1,000)
TR	5,500	4,000	4,000	4,000
TE	96	79	74	74
MATRIX	320 x 320	384 x 384	112 x 112	112 x 112
FOV	240	200	200	200
Slices Thickness	3.5	4.5	4.5	4.5
Acquisition Time	83	83	83	83
Flip Angle	90	90	90	90
